# Research on upper limb rehabilitation assessment model based on belief rule base

**DOI:** 10.3389/fbioe.2024.1469598

**Published:** 2025-01-06

**Authors:** Dawei Jiang, Zixu Zhao, Lijun Wang, Chao Zhang, Meixuan He, Tiejun Ji

**Affiliations:** ^1^ Institute of Technology, Changchun University of Technology, Changchun, China; ^2^ Computer Science and Engineering, Changchun University of Technology, Changchun, China; ^3^ Institute of Applied Technology, Changchun University of Technology, Changchun, China; ^4^ School of Mechanical and Vehicular Engineering, Beijing Institute of Technology, Beijing, China; ^5^ Rehabilitation Department, Jilin Electric Power Hospital, Changchun, China

**Keywords:** upper limb rehabilitation, rehabilitation assessment, belief rule base, expert knowledge, evidential reasoning

## Abstract

Rehabilitation assessments hold an irreplaceable role in the field of rehabilitative therapy. However, due to the subjectivity of traditional physicians and the variability of patient conditions, this leads to a lack of detailed grading and inaccurate assessment results. To address this issue, we developed an upper limb rehabilitation evaluation model. This model integrates muscle strength assessment methods and the Belief Rule Base (BRB), along with qualitative knowledge such as clinical rehabilitation theories and expert experiences. It also utilizes training data from actual patients, collected by an upper limb rehabilitation robot. We then optimized the BRB model’s evaluation accuracy using the Fmincon algorithm and compared its result with commonly used methods such as the Back Propagation (BP) neural network and Support Vector Machine (SVM). This comparison validated the effectiveness and advancement of our BRB approach. This work has laid both a theoretical and practical groundwork for developing a clinical decision support system based on the BRB for upper limb rehabilitation evaluations.

## 1 Introduction

In the field of rehabilitation medicine, assessing the progress and effects of patients’ rehabilitation is a crucial part. However, due to the complex and varied conditions of patients with upper limb impairment, rehabilitation therapists often face the challenge of not being able to accurately diagnose them. For example, in one real work, a stroke sequelae patient sought rehabilitation services due to upper limb dysfunction. Despite partial data obtained through a series of quantitative tests, such as muscle strength tests, the lack of a comprehensive assessment model leaves therapists to rely on personal experience and limited data to make judgments, which often results in less precise or comprehensive assessments.

In order to solve these problems, researchers began to explore various rehabilitation assessment models. At present, these models can be broadly divided into two categories: traditional statistical methods (Meng et al., 2022; Li et al., 2010; Lin et al., 2019) and machine learning methods (Hamaguchi et al., 2020; Tang, 2020; Ahmed et al., 2021; Saibene et al., 2017). Among them, machine learn-based methods have received more and more attention due to their ability to process large amounts of complex data, and have been applied in many fields, including expert systems, neural networks, and so on. However, because each patient’s specific situation is different, building a rehabilitation assessment model that takes into account both quantitative information and the subjective knowledge of experts remains a challenge.

This paper aims to propose a new model that combines muscle strength assessment methods with Belief Rule Base (BRB) theory, in order to provide a more accurate and comprehensive assessment of upper limb rehabilitation effects. Next, we will first introduce the relevant work background, and then elaborate our proposed BRB rehabilitation assessment model and its optimization process.

In recent years, there has been rapid development and application of rehabilitation assessment models for patients. A well-designed rehabilitation assessment model can effectively integrate subjective expert knowledge with uncertain data to evaluate the rehabilitation outcomes of patients. For instance, Das et al. (2013) developed an expert system for hypertension diagnosis using fuzzy rules in 2013, demonstrating high predictive accuracy. In 2016 (Abunaser, 2016), Abu-Naser et al. created an expert system for lower back pain diagnosis and treatment based on rules and knowledge. Furthermore, De La Concepcion et al. (2017) utilized smartphones as detection tools to accurately recognize mobile activity and detect falls in elderly individuals through real data training and movement detection algorithms in 2017. Additionally, Oyelade et al. (2018) established an expert system for breast cancer using NL parser and dictionary database in 2018 to assess the risk of breast cancer in patients through inference models and rule bases.

The aforementioned approach necessitates substantial data support and heavily relies on expert knowledge. However, because expert knowledge is very subjective, it is difficult to establish rehabilitation assessment model only through expert knowledge in many rehabilitation assessment systems. Moreover, these systems that rely on extensive data support and expert knowledge cannot ensure the accuracy of the rehabilitation evaluation model. Therefore, there is a need to develop a rehabilitation evaluation model capable of incorporating quantitative information and expert subjective knowledge while effectively managing complex data and decision-making processes.

The Belief Rule Base (BRB) is a modeling method that integrates quantitative data and expert subjective knowledge to effectively address uncertainty in complex decision-making problems. The concept of BRB was initially introduced by Professor Jianbo Yang (Ribernik and Liu, 2006) from the University of Manchester in 2006. Subsequently, Professor Zhijie Zhou from Rocket Force Engineering University further enriched and developed the BRB theory, proposing an optimized learning method for BRB structure based on the original theory. His contributions to the fundamental theoretical research of BRB parameters and structure iterative learning methods have been significant (Gong et al., 2017; Karim et al., 2017; Chen et al., 2019; Feng et al., 2018). Due to its exceptional decision-making capabilities, the belief rule base has found applications across various fields, fulfilling its corresponding role effectively.

In recent years, numerous scholars have dedicated their efforts to the exploration and advancement of BRB theory, applying it within the realm of medical decision-making. Kong Guilan from Peking University has developed a clinical decision support system grounded in BRB and a clinical assessment decision support system for risk stratification of patients with cardiac chest pain, effectively addressing the uncertainty inherent in clinical domain knowledge and data (Yin et al., 2022; Chang et al., 2019). Saifur Rahaman from the International Islamic University of Chittagong has conducted research on an expert system for diabetes diagnosis based on belief rule database to enhance diagnostic accuracy and reduce costs (Kong et al., 2009). Hossain et al. (2014) utilized an expert system employing belief rule base, gathered real patient data, and diagnosed influenza disease through rule-based reasoning methods. Raihan et al. (2022) employed belief rule base for diagnosing Alzheimer’s disease. Maitri Patel (Patel et al., 2013) and Komal R (Hole and Gulhane, 2013) also utilized belief rule base expert systems for diagnosing symptoms related to memory loss and viral infection respectively.

It is evident that BRB can be utilized to construct an effective model for assessing patients’ recovery status. However, in practical application, it is necessary to pre-divide patients’ conditions based on different diseases in order to enhance the accuracy of sample data provided by the recovery model. Nevertheless, in certain exceptional cases, rehabilitation experts may encounter challenges in accurately determining parameter values due to variations in patients’ physical conditions and diagnostic requirements. This could result in discrepancies between the initial BRB output and actual results, thereby diminishing the precision of upper limb rehabilitation evaluation. As shown in Table 1, the rehabilitation evaluation models have their own advantages and disadvantages. To address these issues, this paper proposes an optimized BRB rehabilitation evaluation model using the Fmincon algorithm. This model establishes more precise sample data through classification of upper limb muscle strength and enhances Evidential Reasoning (ER) of BRB with the Fmincon algorithm to improve the accuracy of rehabilitation evaluation outcomes.

**TABLE 1:** Comparison of rehabilitation assessment models.

Rehabilitation evaluation model	Advantage	Disadvantage
Expert system	The assessment has high precision	Rely on expert knowledge
Rehabilitation evaluation system	Do not need a lot of knowledge	The assessment has low precision
Belief rule base	Accurate use of quantitative knowledge	The assessment results are not detailed enough

The paper is structured as follows: The “Problem Description” section outlines the key issues to be addressed; the “BRB rehabilitation evaluation Model” section presents an optimized BRB rehabilitation evaluation model based on the Fmincon algorithm, with detailed procedural descriptions; in the “experimental verification and comparison” section, experimental validation is conducted and the BRB rehabilitation evaluation model is compared with models established using alternative algorithms. Finally, a conclusion is provided in the “Conclusion” section.

## 2 Description of the problem

In the process of establishing an upper limb rehabilitation assessment model based on BRB, we face several key issues. These issues not only guide our research design but also determine the specific steps and methodological choices for model construction. The following is an overview of the main research questions and their corresponding study designs:

Question 1: How to effectively process the data and classify the evaluation levels for different patients?

In the actual rehabilitation process, we have found that the upper limb muscle strength data of patients is highly variable. Due to differences in the patients’ age, gender, and initial status, the data on their upper limb muscle strength becomes very complex. This greatly affects the ability of rehabilitation therapists to adjust the patients’ rehabilitation training plans. Therefore, handling upper limb rehabilitation data is extremely important. At the same time, we have observed significant differences in the actual performance of patients with different health statuses. The Brunnstrom grading method cannot fully express this variation, which also impacts the ability of rehabilitation therapists to develop tailored rehabilitation plans for patients. Thus, the first question is how to effectively process the data and classify the evaluation levels for different patients.

Question 2: How to improve the accuracy of rehabilitation assessment results?

There are many methods for evaluating rehabilitation, but most of them fail to utilize expert experience and qualitative knowledge effectively, often resulting in low accuracy of rehabilitation assessment outcomes. Since the results of rehabilitation assessments are crucial for therapists to develop subsequent plans for patients, a highly accurate rehabilitation assessment model is essential. Therefore, the second question is how to improve the accuracy of rehabilitation assessment results.

To address the above issues, we designed the following steps: 1. Analysis of upper limb rehabilitation mechanisms and muscle strength assessment; 2. Data collection and preprocessing; 3. Establishment of the BRB model; 4. Model validation and optimization; 5. Practical application testing. Through such a systematic methodological framework, we aim to develop an efficient and reliable tool for evaluating upper limb rehabilitation outcomes, providing strong support for clinical doctors.

## 3 Establish an upper limb rehabilitation assessment model based on BRB

**FIGURE 1:**
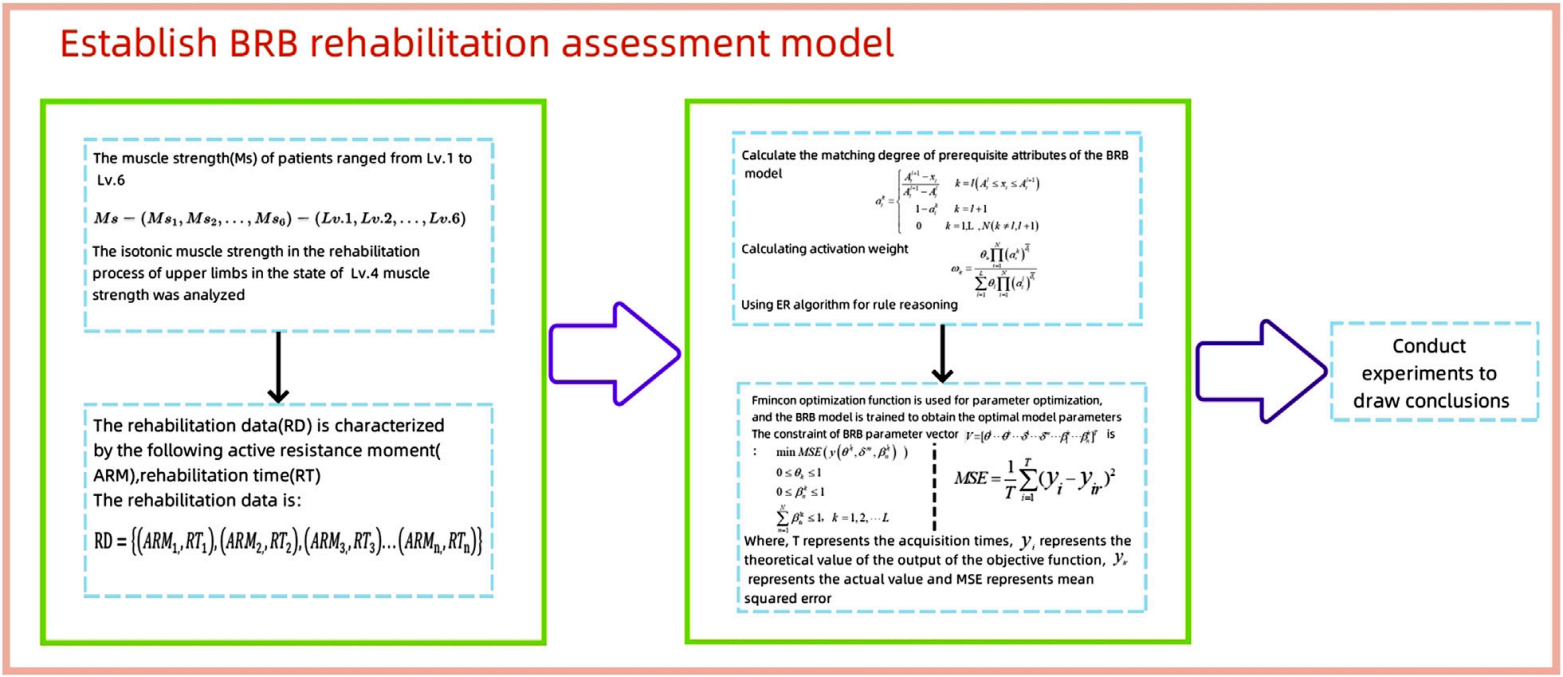
Establish a flow chart of BRB rehabilitation assessment model.

The establishment of BRB rehabilitation assessment model needs to analyze the rehabilitation mechanism to obtain the sample data of rehabilitation assessment and optimize the model to achieve better rehabilitation assessment effect. Figure 1 shows the process of establishing the BRB rehabilitation assessment model.

Firstly, the muscle strength data were graded. The active resistance moment (ARM) and rehabilitation time (RT) were sorted into rehabilitation data (RD). The prerequisite attribute weight and activation weight of BRB were calculated. Using ER rules for inference, the Fmincon optimization function and MSE (mean square error) function were employed for parameter optimization. The BRB model was then trained to obtain the optimal model parameters under BRB parameter vector constraints.

### 3.1 Analysis of upper limb rehabilitation mechanism

This paper mainly analyzes the maximum resistance that the isotonic muscle strength can overcome when the knuckle moves in full amplitude in the process of upper limb rehabilitation in the state of grade 4 muscle strength. Through the upper limb rehabilitation training robot, the muscle strength data of patients in the active rehabilitation process was collected in real time and evaluated by scientific evaluation method. This assessment includes the Maximum resistance to do a Repetition, called an I Repetition Maximum (IRM), and the maximum resistance to overcome at the completion of 10 repetitions (10 IRM). Assessment results can be used by rehabilitation therapists in the following ways:

1. Aid rehabilitation therapists in assessing the current upper limb strength of patients.
2. Assist rehabilitation therapists in determining the extent of upper limb nerve injury.
3. Support rehabilitation therapists in identifying the next steps for rehabilitation treatment.
4. Assist rehabilitation therapists in evaluating the effectiveness of early rehabilitation interventions.

Therefore, by continuously collecting real-time muscle strength data and other relevant rehabilitation information, and integrating it with the expertise of rehabilitation therapists, this study will incorporate a method for assessing muscle strength into an upper limb rehabilitation training robot. This method will categorize data, establish a muscle strength database for different patient profiles, and provide tailored resistance levels and scientifically-based muscle strength ratings. The grading system for upper limb muscle strength is presented in Table 2 (Naghdi et al., 2010).

**TABLE 2:** Upper limb muscle strength grading table.

Serial number	Muscle strength level	Evaluation criteria
1	Lv.1	There was no sign of muscle contraction
2	Lv.2	There was muscle contraction, but no joint movement
3	Lv.3	Remove the influence of gravity on the limbs, and the joints can move to the maximum range
4	Lv.4	Against the gravity of the limb itself, the joint is able to move to its maximum range
5	Lv.5	Maximum range of motion can be achieved under moderate resistance
6	Lv.6	Maximum range of motion can be achieved with sufficient resistance

### 3.2 Feature quantity acquisition

**FIGURE 2:**
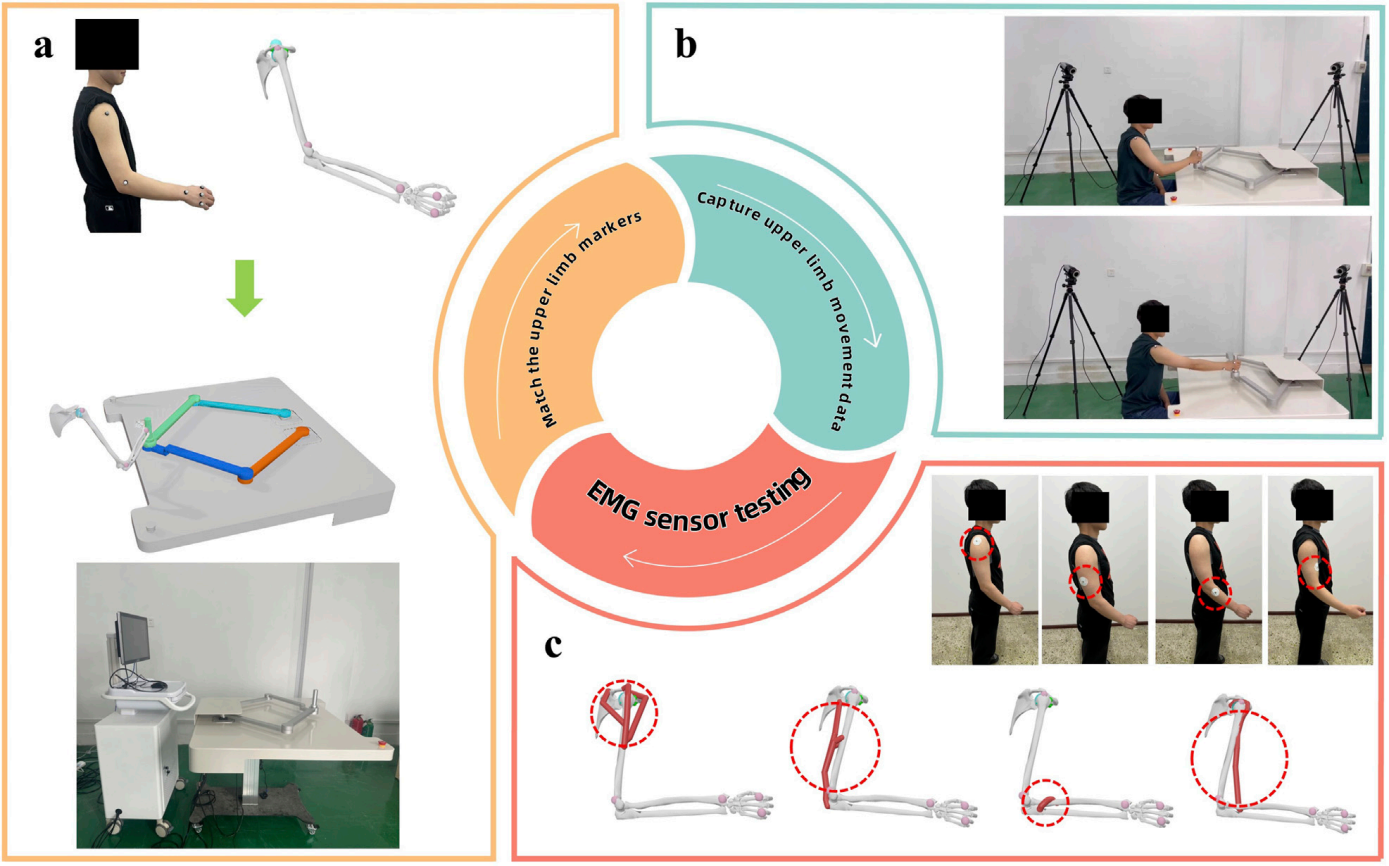
Upper limb feature data acquisition map.

The input parameters for this study primarily encompass basic patient information and upper limb characteristic data collected via the rehabilitation robot. Specifically: Basic patient information includes age, gender, medical history, etc. Upper limb characteristic data: Collected using the upper limb robot system shown in Figure 2, covering health indicators such as active resistance torque and recovery time. These data are obtained through methods like marker matching, motion capture technology, and electromyography (EMG) sensor testing.

To ensure the accuracy and reliability of the research results, all patients participating in this experiment must meet the following criteria: 1. Able to independently complete specified movements during moderate-intensity upper limb muscle strength training; 2. The onset time is between 1 week to 3 months; 3. Age range is between 20 and 70 years old; 4. No history of cognitive impairment or neurological diseases such as epilepsy; 5. All patients have signed informed consent forms.

Based on the aforementioned patient criteria, all subjects in this study met the Level 5 requirements of the Brunnstrom scale. Consequently, three different experiments were designed with varying levels of active resistance torque for the upper limbs, denoted as E1, E2, and, E3. The specific experiments are shown in Table 3. It is assumed that under these three sets of experiments, the health statuses of the patients’ upper limbs correspond to First-degree Fault, Second-degree Fault, Third-degree Fault, and Fourth-degree Fault, respectively.

**TABLE 3:** Patients rehabilitation experiment table.

Experimental number	Active resistance torque for the upper limbs	Action times
E1	5	50
E2	8	50
E3	11	50

In the table, each experiment represents the patient’s rehabilitation training action under the specified upper limb active resistance moment. Each action is completed, and the number of actions is recorded as 1. When the patient completes 50 actions, it indicates that the patient has completed the experiment.

**FIGURE 3:**
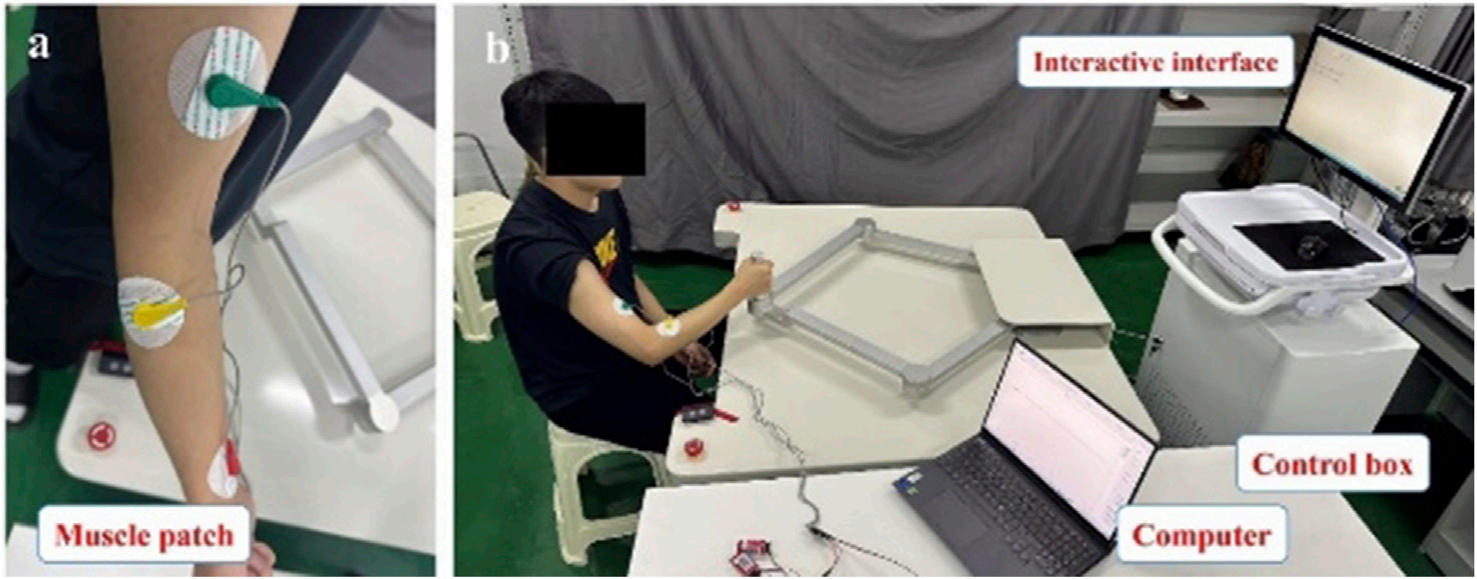
Diagram of the patient’s upper limb training.

During the experimental process, muscle patches were affixed to the upper limbs of each patient, as illustrated in Figure 3. Daily training sessions involving E1, E2, and E3 were conducted based on the patients’ rehabilitation status.

The upper limb feature data collected by the rehabilitation robot will be used as the input of the BRB model, that is, the prerequisite attribute. By combining these characteristic quantities, confidence rules can be formed to infer the recovery status of the patient’s upper limb. In this process, the qualitative knowledge of the rehabilitation physician can be used to determine the importance and reference values of characteristic quantities such as active resistance moment and recovery time, and serve as inputs to the BRB model. Therefore, BRB theory can be effectively applied to the evaluation of upper limb rehabilitation status. On the basis of in-depth study of BRB theory system, this paper focuses on the evaluation and prediction of upper limb rehabilitation of patients, and expands the application of BRB theory in the assessment of upper rehabilitation status.

### 3.3 Design of upper limb rehabilitation evaluation model based on BRB

**FIGURE 4:**
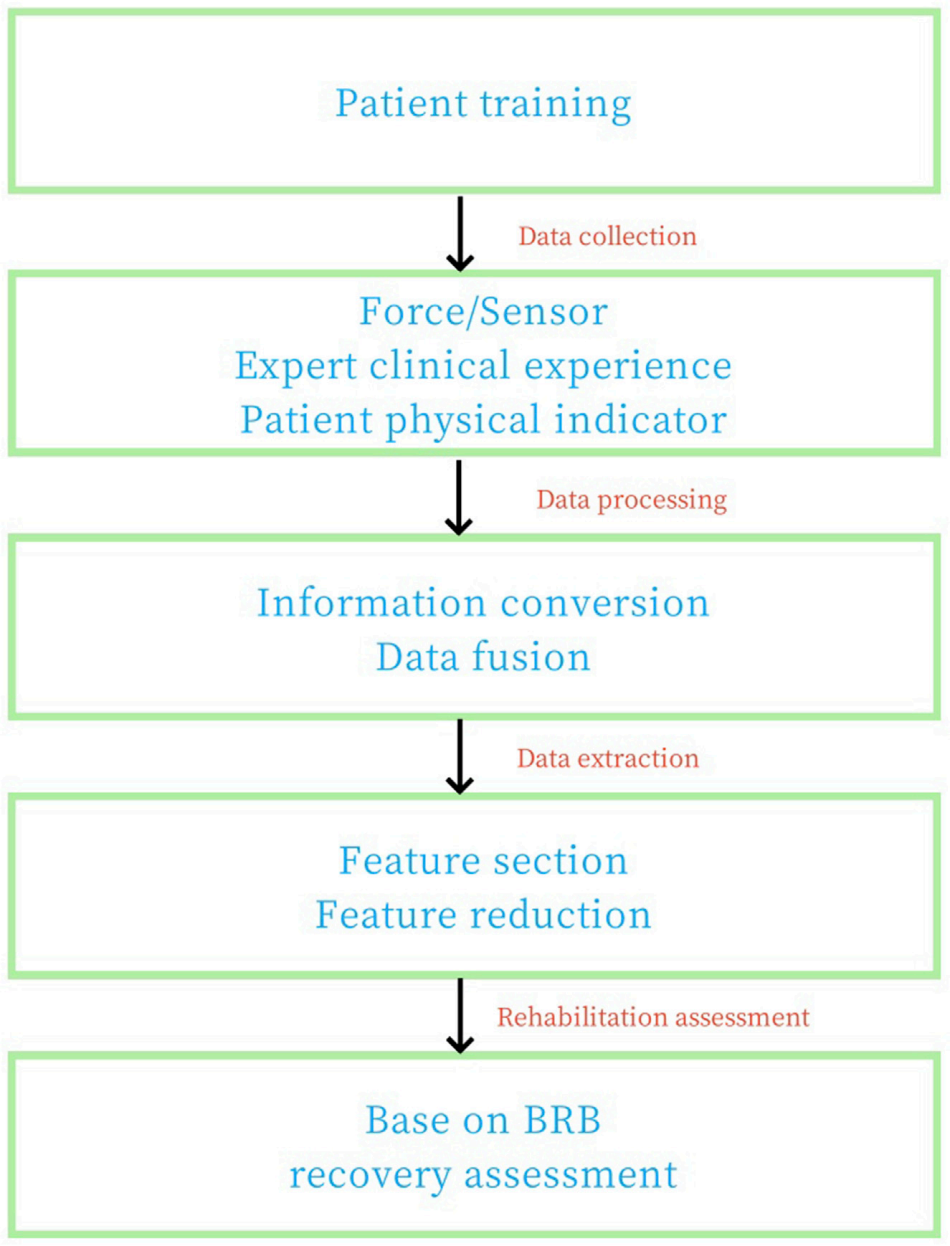
Block diagram of rehabilitation effect evaluation of human upper limb based on BRB model.

The evaluation block diagram of the human upper limb rehabilitation effect based on BRB is shown in Figure 4. First of all, data collection was carried out in Jilin Electric Power Hospital to collect real and effective quantitative test data and expert qualitative knowledge. Based on quantitative rehabilitation evaluation indicators, constraint conditions of characteristic quantity were set up, combined with rehabilitation exercise mechanism, and on the basis of quantitative rehabilitation evaluation parameters, a rehabilitation degree evaluation model was established by using beleif rule base theory.

In order to evaluate the rehabilitation state of the patient’s upper limb, this section will establish the relationship between the health state of the patient’s upper limb and the characteristic quantity based on the BRB theory by using the nonlinear relationship model f. The specific relationship model is shown in Formula 1:

$$H=f\left(\begin{array}{c} x_{1t},x_{2t},\ldots .,x_{Mt},V \end{array}\right)$$ (1)

where H represents the health state of the patient’s upper limb predicted by the BRB model; x represents the characteristic quantity of the evaluation system; f Represents nonlinear function; V represents the weight parameters set inside the model. The characteristic quantity includes the characteristic quantity, such as the active resistance moment and active recovery time collected by the system.

In the process of establishing the patient’s upper limb health assessment model based on BRB, firstly, the feature quantity representing the patient’s upper limb health state is extracted, and the feature quantity of the upper limb health state is segmented and used as the input of the BRB model. Secondly, the parameter optimization model of BRB is established, and the parameters of the model are optimized and updated by computer algorithm to improve the accuracy of BRB model in predicting the rehabilitation state of upper limbs. Finally, the rehabilitation status of patients’ upper limbs was evaluated through the trained model.

In the BRB model of patient upper limb health assessment, rule k of BRB is shown in Formula 2:

$$R_{k}:if\;x_{1}\;is\;A_{1}^{k}\wedge x_{2}\;is\;A_{2}^{k}\;\wedge \cdots \wedge x_{M}\;is\;A_{M}^{k}\;$$ (2)

$$Then\left\{\left(D_{1},\beta_{1,k}\right),\left(D_{2},\beta_{2,k}\right),\ldots ,\left(D_{N},\beta_{N,k}\right)\right\}$$

$$with\;a\;new\;rule\;weight\;\theta_{k}\;and\;attribute\;weight\;\delta_{1},\delta_{2},\ldots ,\delta_{M}$$

where $x_{i\left(1,2,\ldots ,M\right)}$ is BRB input, that is, the quantity of the patient’s upper limb health characteristics collected by the upper limb rehabilitation robot; k indicates the number of BRB rules; $A_{i}^{k}\left(1,2,\ldots ,M\right)$ represents the reference value of the *i*th premise attribute; $R_{k}$ represents the *k*th evaluation result of the rule; M and N represent the number of prerequisite attributes and the number of evaluation results respectively; $\beta_{j,k}$ represents the belief of the first evaluation result j; $\theta_{k}$ represents the weight of rule k, and represents the weight of the $\delta_{i}$ epresents the weight of the *i*th premise attribute.

### 3.4 ER-based rule inference process

In the process of BRB rule Reasoning, Evidential Reasoning (ER) algorithm is used to combine the confidence rules to get the final system output, which is the beleif rule base reasoning method based on evidence reasoning algorithm (Belief Rule-base Inference Methodology Using the Evidential Reasoning Approach) (Yang et al., 2006; Yang and Singh, 1994). The whole reasoning process consists of three main steps (Zhijie et al., 2017).

Step 1: calculate the matching degree of the prerequisite attribute of the belief rule base model, that is, the matching degree of the feature quantity. The matching degree reflects the matching degree between the feature quantity and the rule. The formula for calculating the matching degree of the prerequisite attribute of rule k is shown in Formula 3:

$$a_{i}^{k}=\left\{\begin{array}{cc} \frac{A_{i}^{l+1}-x_{i}}{A_{i}^{l+1}-A_{i}^{l}}\; & k=l\left(A_{i}^{l}\leq x_{i}\leq A_{i}^{l+1}\right)\\ 1-a_{i}^{k}\; & k=l+1\\ 0\; & k=1,\;L,N\left(k\neq l,l+1\right) \end{array}\right.$$ (3)

where, $a_{i}^{k}$ represents the matching degree of the i premise attribute in rule k; $A_{i}^{l}$ and $A_{i}^{l+1}$ represent the i - th prerequisite attribute reference value in the two adjacent rules, respectively.

Step 2: Calculate the activation weight.

This process is used to determine which rules should be activated and considered during reasoning, better reflecting the uncertainties and fuzziness in expert knowledge, thereby improving the model’s adaptability to unknown situations to further determine the final output of the system. The degree to which different rules are activated depends on how well they match the input data. According to the BRB model, in order to facilitate the understanding and interpretation of the decision-making process of the model, the setting of the activation weights should be transparent and adjustable, and the relative relationship between them should be consistent. By calculating the activation weights, the contribution degree of different rules can be evaluated, and the reasoning basis based on the matching degree can be provided for the system. The formula for calculating the activation weight of Rule k is shown in Formula 4:

$$\omega_{k}=\frac{\theta_{k}\overset{N}{\underset{i=1}{\prod }}{\left(a_{i}^{k}\right)}^{{\bar{\delta }}_{i}}}{\overset{L}{\underset{l=1}{\sum }}\theta_{l}\overset{N}{\underset{i=1}{\prod }}{\left(a_{i}^{l}\right)}^{{\bar{\delta }}_{i}}}$$ (4)

where ${\bar{\delta }}_{i}$ indicates attribute weight; $a_{i}^{k}$ represents the matching degree of the input feature quantity relative to the *i*th attribute; $\theta_{k}$ represents the corresponding rule weight.

Step 3: Use the ER algorithm for rule reasoning.

ER algorithm can deal with the combination of expert knowledge, deal with uncertainty, and is suitable for a variety of scenarios. ER algorithm can effectively synthesize the inference results of multiple rules, considering the weight and correlation between them, so as to reach a more accurate conclusion. By using the ER algorithm, the limitation of single-rule reasoning can be overcome, and the performance and effect of the system in decision-making and evaluation tasks can be improved. All rules in the BRB model are reasoned and analyzed by using the ER algorithm, and the final output result is obtained as shown in Formula 5:

$$S\left(x\right)=\left\{\left(D_{j},{\hat{\beta }}_{j}\right),\;j=1,2,\ldots ,L,N\right\}$$ (5)

where, $\hat{\beta_{j}}$ indicates the belief degree of the evaluation result $D_{j}$ , as shown in Formula 6:

$${\hat{\beta }}_{j}=\frac{\mu \times \left[\overset{L}{\underset{k=1}{\prod }}\left(\omega_{k}\beta_{j,k}+1-\omega_{k}\overset{N}{\underset{i=1}{\sum }}\beta_{i,k}\right)-\overset{L}{\underset{k=1}{\prod }}\left(1-\omega_{k}\overset{N}{\underset{i=1}{\sum }}\beta_{i,k}\right)\right]}{1-\mu \times \left[\overset{L}{\underset{k=1}{\prod }}\left(1-\omega_{k}\right)\right]}$$ (6)

Formula 7 serves as a regulator in the ER model handling process, responsible for adjusting the evaluation result model. When μ increases, the evaluation result will be more conservative; when μ decreases, the evaluation result will be more aggressive.

$$\mu ={\left[\overset{N}{\underset{j=1}{\sum }}\overset{L}{\underset{k=1}{\prod }}\left(\omega_{k}\beta_{j,k}+1-\omega_{k}\overset{N}{\underset{i=1}{\sum }}\beta_{i,k}\right)-\left(M-1\right)\overset{L}{\underset{k=1}{\prod }}\left(1-\omega_{k}\overset{N}{\underset{i=1}{\sum }}\beta_{i,k}\right)\right]}^{-1}$$ (7)

where N represents the number of evaluation results; $\hat{\beta_{j}}$ Is a function of rule weights $\theta_{k}$ , attribute weights $\bar{\delta_{i}}$ , and belief $\beta_{j,k}$ .

Assuming that $D_{j}$ the utility of the evaluation result is $\mu \left(D_{j}\right)$ , then the expected utility is S(X) as shown in Formula 8:

$$\mu \left(S\left(X\right)\right)=\overset{M}{\underset{j=1}{\sum }}\mu \left(D_{j}\right)\beta_{j}$$ (8)

where $\beta_{j}$ indicates the belief of the output relative to $D_{j}$ .

Therefore, the output of the BRB-based health assessment model $\hat{y}$ is expressed as shown in Formula 9:

$$\hat{y}=\mu \left(S\left(X\right)\right)$$ (9)

### 3.5 BRB parameter optimization based on fmincon algorithm

In general, the rehabilitation specialist gives the initial BRB parameters based on the collection of historical information and empirical knowledge of the patient’s upper limb status. However, in special cases, it is difficult for rehabilitation specialists to accurately determine the value of these parameters due to differences in the patient’s physical condition and diagnostic requirements. This can lead to deviations between the initial BRB output and the actual results, thus reducing the accuracy of the evaluation of the upper limb rehabilitation effect. Fmincon is a nonlinear multivariable function with constraints Sreeraj (2013), which is often used to solve the minimum value of a nonlinear multivariate function. Therefore, this paper adopts Fmincon as an optimization function, takes mean square error as the input objective function for parameter optimization, and trains the BRB model to obtain the optimal model parameters. To minimize the error between the actual output result and the initial BRB output result, improve the evaluation accuracy of upper limb rehabilitation effect.

In the process of parameter optimization of BRB rehabilitation evaluation model, the following optimization objective function is established as shown in Formula 10:

$$\begin{array}{c} Ms=\left[\theta^{1}\cdots \theta^{k}\cdots \delta^{1}\cdots \delta^{m}\cdots \beta_{1}^{k}\cdots \beta_{n}^{k}\right]T\\ \min MSE\left(y\left(\theta^{k},\delta^{m},\beta_{n}^{k}\right)\right)\\ 0\leq \theta_{k}\leq 1\\ 0\leq \beta_{n}^{k}\leq 1\\ \overset{N}{\underset{n=1}{\sum }}\beta_{\text{n}}^{\text{k}}\leq 1,\;k=1,2,\cdots L \end{array}$$ (10)

Where as shown in Formula 11:

$$MSE=\frac{1}{T}\overset{T}{\underset{i=1}{\sum }}{\left(y_{i}-y_{ir}\right)}^{2}$$ (11)

where, T represents the rehabilitation data volume, $y_{i}$ represent output of rehabilitation evaluation model for patients, and $y_{ir}$ represent output results of actual rehabilitation state of patients.

### 3.6 Establishment of BRB health assessment model

In the process of establishing the health assessment model of upper limb active rehabilitation, it is very important to select the reference index of upper limb rehabilitation state. According to the communication of rehabilitation experts in Jilin Electric Power Hospital, combined with the muscle strength sensor of rehabilitation robot. Finally, the active resistance torque of upper limb (ARTUL), Rehabilitation Training Time (RTT) and Mean Amplitude of Muscle Strength (MAMS) were selected as the reference indexes of upper limb rehabilitation, and three reference values were selected for every three reference indexes. These indicators will be used as the main basis for evaluating the health status of upper limbs, and verified by simulation verification data. In order to verify the evaluation model, because the main research object of this paper is the patients with grade 5 in Brunnstrom muscle strength table, after patient screening and training, 40 patients with grade 5 in Brunnstrom muscle strength table are finally determined for follow-up testing. According to the actual training of patients and communication with rehabilitation experts, Brunnstrom muscle strength is subdivided into 4 grades again for evaluation example verification as shown in formula 15.

For ARTUL, take 3 reference values, Small, Normal and Big, denoted by S , N and B , respectively as shown in Formula 12:

$$A_{1}^{k}\in \left\{S,N,B\right\}$$ (12)

Similarly, for RTT, take 3 reference values, Small, Normal and Long, denoted by S , N and L , respectively as shown in Formula 13:

$$A_{2}^{k}\in \left\{S,N,L\right\}$$ (13)

Similarly, for MAMS, take 3 reference values, Small, Normal and High, denoted by S , N and H , respectively as shown in Formula 14:

$$A_{3}^{k}\in \left\{S,N,H\right\}$$ (14)

As for the evaluation results, it can be seen from the experiment that there are 4 health states, respectively represented as I,II, III and IV, namely as shown in Formula 15:

$$D=\left(\begin{array}{ccccc} D_{1} & D_{2} & D_{3} & D_{4} & \end{array}\right)=\left(I\;II\;III\;IV\right)$$ (15)

The input feature quantity of the BRB has 3 reference indicators, and each reference indicator has 3 reference values, from which 27 initial confidence rules can be established to evaluate the health state of the system. Taking ARTUL as S as an example, the following rules are established according to expert knowledge as shown in Formula 16:

$$R_{1}:If\;\left(ATRUL\;is\;S\right)\;\wedge \left(RTT\;is\;S\right)\wedge \left(MAMS\;is\;S\right),Then\left\{\left(1,0,0,0,0\right)\right\}$$

$$R_{2}:If\;\left(ATRUL\;is\;S\right)\;\wedge \left(RTT\;is\;S\right)\wedge \left(MAMS\;is\;N\right),Then\left\{\left(0.9,0.1,0,0,0\right)\right\}$$

$$R_{3}:If\;\left(ATRUL\;is\;S\right)\;\wedge \left(RTT\;is\;S\right)\wedge \left(MAMS\;is\;H\right),Then\left\{\left(0.8,0.2,0,0,0\right)\right\}$$

$$R_{4}:If\;\left(ATRUL\;is\;S\right)\;\wedge \left(RTT\;is\;N\right)\wedge \left(MAMS\;is\;S\right),Then\left\{\left(0.9,0.1,0.0,0,0\right)\right\}$$

$$R_{5}:If\;\left(ATRUL\;is\;S\right)\;\wedge \left(RTT\;is\;N\right)\wedge \left(MAMS\;is\;N\right),Then\left\{\left(0.8,0.2,0,0,0\right)\right\}$$

$$R_{6}:If\;\left(ATRUL\;is\;S\right)\;\wedge \left(RTT\;is\;N\right)\wedge \left(MAMS\;is\;H\right),Then\left\{\left(0.7,0.3,0,0,0\right)\right\}$$

$$R_{7}:If\;\left(ATRUL\;is\;S\right)\;\wedge \left(RTT\;is\;L\right)\wedge \left(MAMS\;is\;S\right),Then\left\{\left(0.6,0.3,0.1,0,0\right)\right\}$$

$$R_{8}:If\;\left(ATRUL\;is\;S\right)\;\wedge \left(RTT\;is\;L\right)\wedge \left(MAMS\;is\;N\right),Then\left\{\left(0.5,0.4,0.1,0,0\right)\right\}$$

$$R_{9}:If\;\left(ATRUL\;is\;S\right)\;\wedge \left(RTT\;is\;L\right)\wedge \left(MAMS\;is\;H\right),Then\left\{\left(0,0.9,0.1,0,0\right)\right\}$$ (16)

Taking the above rules as an example, a systematic BRB rehabilitation evaluation model is established. The *k*th rule is as shown in Formula 17:

$$R_{k}:if\;ATRUL\;is\;A_{1}^{k}\;\wedge RTT\;is\;A_{2}^{k}\wedge MAMS\;is\;A_{3}^{k},$$ (17)

$$Then\;Health-condition\;is\;\left\{\left(I,\beta_{1,k}\right),\left(II,\beta_{2,k}\right),\left(III,\beta_{3,k}\right),\left(IV,\beta_{4,k}\right)\right\}$$

$$with\;rule\;weight\;\theta_{k}\;and\;attribute\;weight\;\delta_{1},\delta_{2},\ldots ,\delta_{4}$$

## 4 Verification and analysis

### 4.1 Experimental verification

The validity of the health assessment model established in this section is verified by simulation analysis. During the experiment, 2,000 groups of characteristic values in three states were collected, of which 1,600 groups were used as training sets and 400 groups as test sets.

First of all, according to the reference values given by the rehabilitation therapist of Jilin Electric Power Hospital and the analysis of the characteristics of the sampled data, the semantic reference values of the characteristic quantities and evaluation results are quantified, as shown in Tables 4–6.

**TABLE 4:** Attribute reference value of ARTUL.

Semantic values	S	N	B
Quantized values	4	8	12

**TABLE 5:** Attribute reference value of RTT.

Semantic values	S	N	L
Quantized values	5	10	15

**TABLE 6:** Attribute reference value of MAMS.

Semantic values	S	N	H
Quantized values	5	8	11

In the rehabilitation evaluation model, since each feature has 3 reference values, there are a total of 27 rules when evaluating the health status of these features. According to expert knowledge, the initial confidence of the three characteristic quantities is shown in Table 7.

**TABLE 7:** Initial parameters given by rehabilitation experts for BRB model.

Number of rules	ARTUL, RTT, MAMS	Failure degree distribution{D1 D2 D3 D4} = {1 2 3 4}
1	S, S, S	{1 0 0 0}
2	S, S, N	{0.9 0.1 0 0}
3	S, S, H	{0.8 0.2 0 0
4	S, N, S	{0.9 0.1 0 0
5	S, N, N	{0.8 0.2 0 0}
6	S, N, H	{0.7 0.3 0 0}
7	S, L, S	{0.6 0.3 0.1 0}
8	S, L, N	{0.5 0.4 0.1 0}
9	S, L, H	{0 0.9 0.1 0}
10	N, S, S	{0.8 0.2 0 0}
11	N, S, N	{0.4 0.5 0.1 0}
12	N, S, H	{0.3 0.4 0.3 0}
13	N, N, S	{0.2 0.6 0.2 0}
14	N, N, N	{0.1 0.2 0.7 0}
15	N, N, H	{0 0.3 0.7 0}
16	N, L, S	{0 0.1 0.9 0}
17	N, L, N	{0 0 1 0}
18	N, L, H	{0 0 0.9 0.1}
19	B, S, S	{0 0.3 0.6 0.1}
20	B, S, N	{0 0.2 0.7 0.1}
21	B, S, H	{0 0.1 0.8 0.1}
22	B, N, S	{0 0 0.7 0.3}
23	B, N, N	{0 0 0.3 0.7}
24	B, N, H	{0 0 0.1 0.9}
25	B, L, S	{0 0 0.2 0.8}
26	B, L, N	{0 0 0.1 0.9}
27	B, L, H	{0 0 0 1}

**FIGURE 5:**
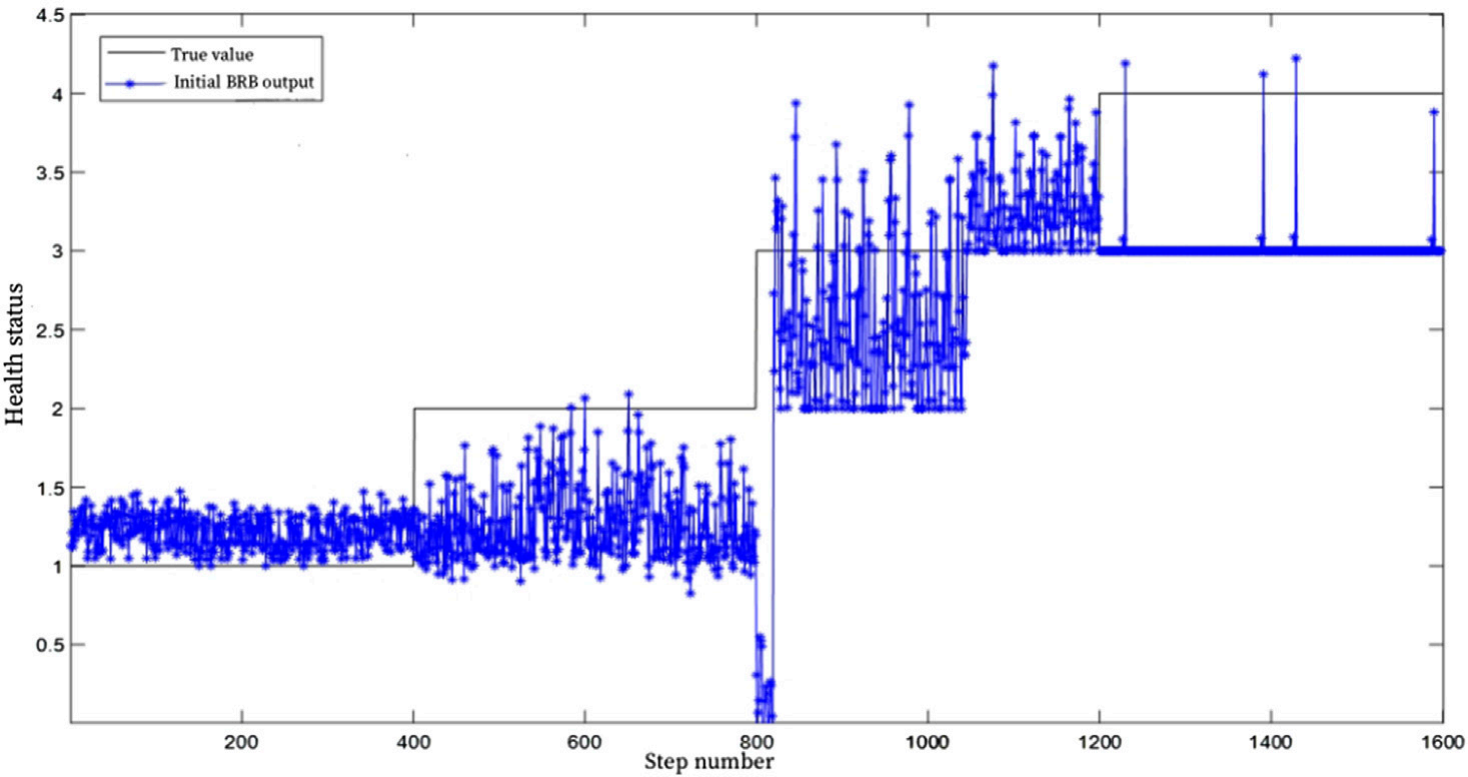
Health assessment results of the initial BRB.

According to the initial parameters given by the experts, regardless of the weight of the characteristic quantity $\delta_{i}$ and $\theta_{k}$ set to 1, the health evaluation result can be obtained, as shown in Figure 5. In Figure 5, the blue distribution represents the initial BRB health assessment results, and black represents the true health assessment results. As can be seen from the figure, the degree of fitting between the evaluation results and the training data is not high.

**FIGURE 6:**
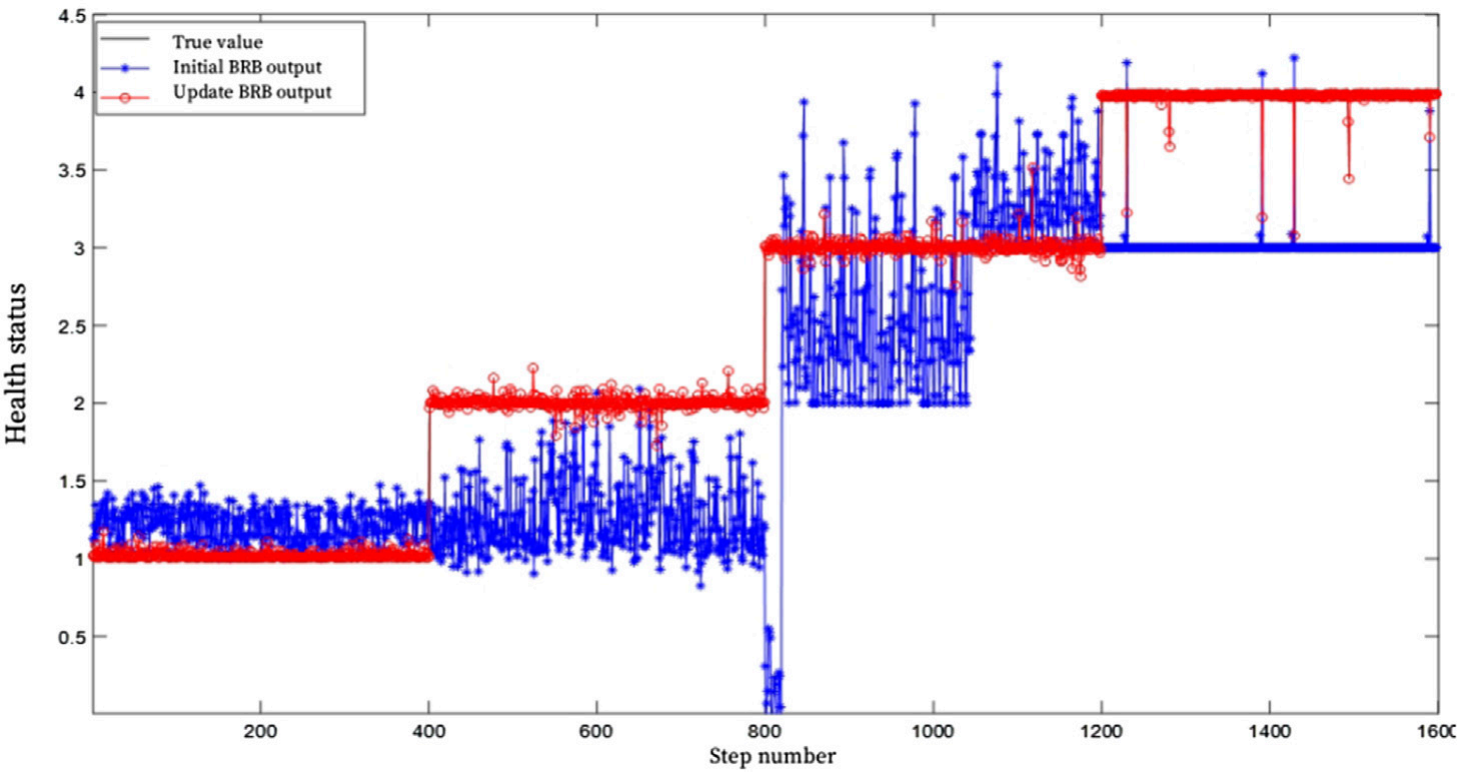
BRB health assessment results after training.

In order to improve the evaluation accuracy of the evaluation model, the parameters of the initial BRB were updated based on the established optimization model and the Fmincon optimization algorithm. The BRB parameters after training are shown in Table 8, and the evaluation results are shown in Figure 6. As can be seen from Figure 6. In Figure 6, the blue distribution represents the initial BRB health assessment results, black represents the real health assessment results, and red represents the BRB health assessment results optimized by Fmincon function, with an accuracy of 92.6%. It can be seen that the BRB health assessment results optimized by Fmincon function have a higher accuracy.

**TABLE 8:** Updated BRB model parameters.

Number of rules	ARTUL, RTT, MAMS	Failure degree distribution{D1 D2 D3 D4} = {1 2 3 4}
1	S, S, S	{0.835 0.15 0.013 0.000}
2	S, S, N	{0.814 0.176 0.010 0.000}
3	S, S, H	{0.723 0.237 0.030 0.010}
4	S, N, S	{0.812 0.117 0.064 0.007}
5	S, N, N	{0.837 0.105 0.047 0.011}
6	S, N, H	{0.643 0.348 0.008 0.001}
7	S, L, S	{0.489 0.377 0.103 0.031}
8	S, L, N	{0.466 0.279 0.228 0.027}
9	S, L, H	{0.027 0.813 0.132 0.028}
10	N, S, S	{0.774 0.196 0.030 0.000}
11	N, S, N	{0.496 0.386 0.107 0.011}
12	N, S, H	{0.319 0.376 0.245 0.060}
13	N, N, S	{0.197 0.631 0.123 0.049}
14	N, N, N	{0.089 0.217 0.597 0.097}
15	N, N, H	{0.013 0.321 0.564 0.102}
16	N, L, S	{0.003 0.064 0.877 0.056}
17	N, L, N	{0.032 0.005 0.899 0.064}
18	N, L, H	{0.001 0.006 0.900 0.093}
19	B, S, S	{0.010 0.280 0.632 0.078}
20	B, S, N	{0.002 0.121 0.796 0.081}
21	B, S, H	{0.006 0.012 0.843 0.139}
22	B, N, S	{0.004 0.053 0.698 0.245}
23	B, N, N	{0.003 0.027 0.325 0.645}
24	B, N, H	{0.003 0.064 0.182 0.751}
25	B, L, S	{0.006 0.009 0.311 0.674}
26	B, L, N	{0.001 0.003 0.082 0.914}
27	B, L, H	{0.007 0.021 0.036 0.936}

### 4.2 Comparative analysis

**FIGURE 7:**
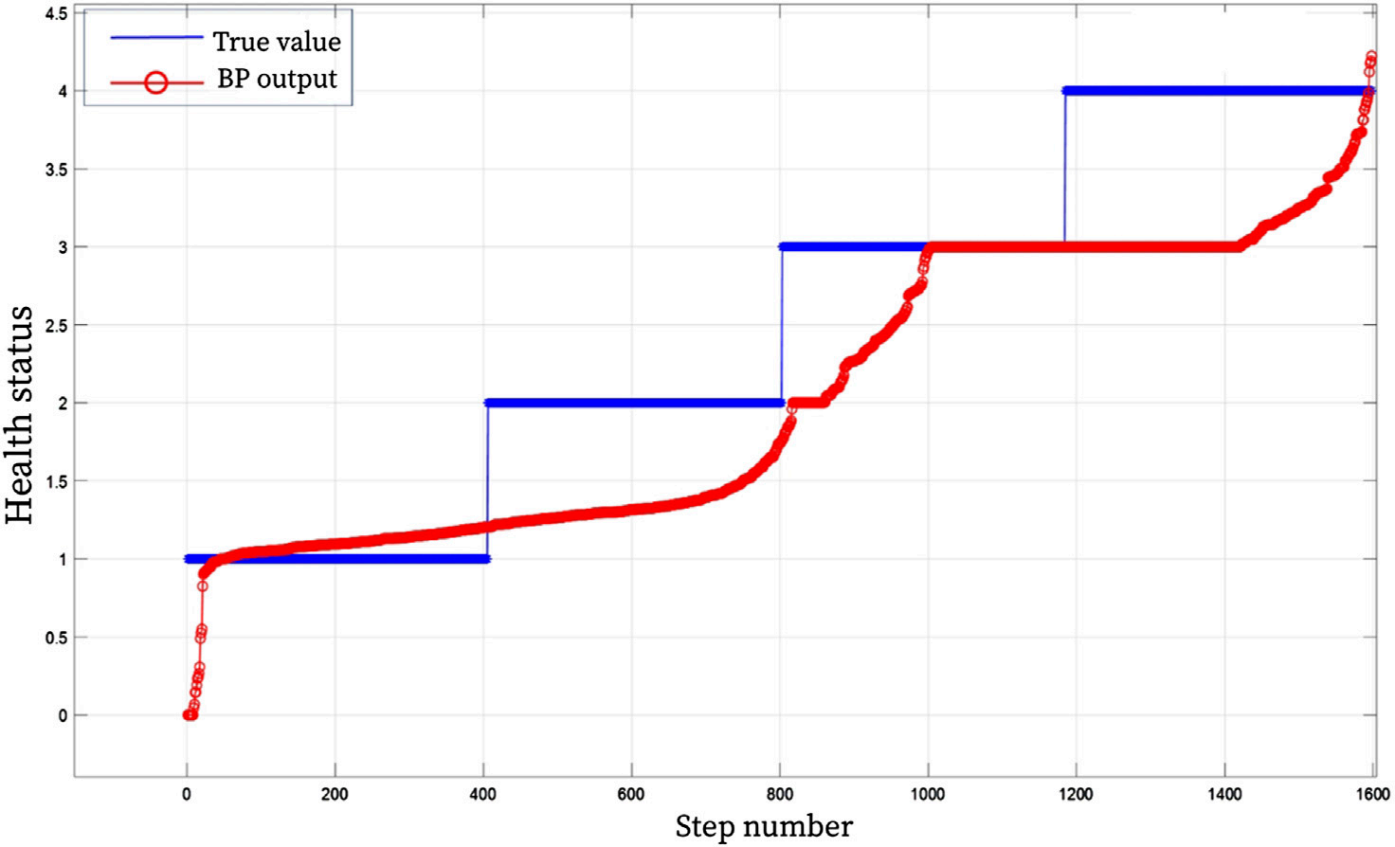
BP health assessment result graph.

**FIGURE 8:**
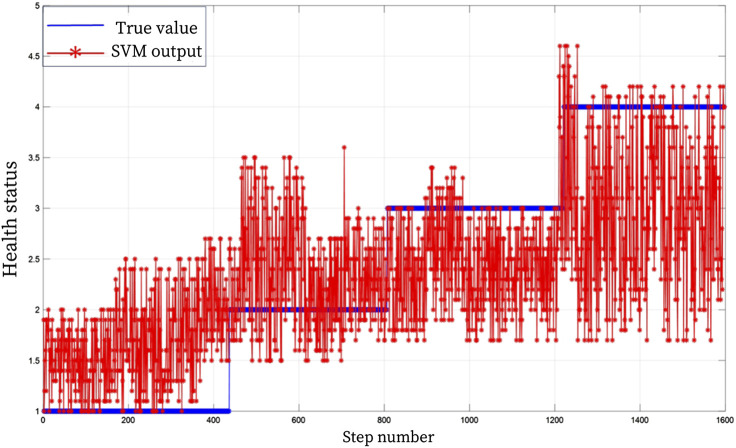
SVM health assessment result graph.

In order to further verify the advanced nature of the proposed model, BP and SVM were used for comparative analysis. BP and SVM algorithms are widely used in diagnosis, classification, prediction and other problems. Similar to BRB model simulation analysis, the SVM and BP algorithm evaluation model were trained and tested 1:1 with 1,600 sets of data. Figures 7, 8 show the results of the rehabilitation degree evaluation model based on support vector machine SVM and BP. It can be observed from the figure that the accuracy of BP neural network in the evaluation of upper limb health status is low for grade 2 and grade 4 health status, and the accuracy of overall rehabilitation evaluation is only 67%, while the overall evaluation stability is poor when SVM evaluates upper limb health status, and the assessed health status fluctuates poorly, and the evaluation accuracy is only 53%. Through the analysis of experimental results, it can be seen that the BRB-based upper limb health evaluation model of patients proposed in this chapter has higher accuracy and effectiveness than the rehabilitation evaluation model based on SVM and BP algorithm.

## 5 Conclusion

Based on the analysis of the evaluation method of upper limb muscle strength and BRB method, a rehabilitation degree evaluation model based on beleif rule base theory (BRB) was established by combining clinical rehabilitation theory, expert clinical experience and collected real and effective quantitative test data of upper limb rehabilitation status. In order to improve the evaluation accuracy of the model and reduce the subjectivity due to expert experience and other qualitative knowledge, Fmincon optimization algorithm was used to optimize the average resistance moment weight, average amplitude weight of muscle force and time weight of BRB. The optimized BRB upper limb rehabilitation evaluation model was compared with the evaluation results of BP neural network and support vector machine algorithm. The accuracy of BRB upper limb rehabilitation evaluation results was 92.6%, which was better than 67% and 53% of BP and SVM. The correctness and advanced nature of BRB assessment method are verified, which lays a theoretical and practical foundation for the establishment of BRB-based clinical upper limb rehabilitation evaluation decision support system.

## Data Availability

The raw data supporting the conclusions of this article will be made available by the authors, without undue reservation.
